# Onabotulinum Toxin A Intradetrusor Injections in Children with Neurogenic Lower Urinary Tract Dysfunction: Long-Term Histological Effects on the Bladder Wall

**DOI:** 10.3390/biomedicines11051300

**Published:** 2023-04-27

**Authors:** Chiara Pellegrino, Valentina Forlini, Federica Lena, Maria Luisa Capitanucci, Francesca Diomedi Camassei, Enrico Castelli, Giovanni Mosiello

**Affiliations:** 1 Division of Neuro-Urology, Bambino Gesù Children’s Hospital, Piazza di Sant’Onofrio, 4, 00165 ERN eUROGEN Affiliated Center, 00118 Rome, Italy; 2 Pediatric Surgery Division, University of Genova, via Balbi 5, 16126 Genoa, Italy; 3 Pathology Unit, Department of Laboratories, Bambino Gesù Pediatric Hospital IRCCS, 00118 Rome, Italy; 4 Pediatric Neurorehabilitation, Bambino Gesù Children’s Hospital, 00118 Rome, Italy

**Keywords:** Onabotulinum Toxin A, neurogenic lower urinary tract dysfunction, neurogenic detrusor overactivity, pediatric urology, bladder fibrosis, bladder histology, spina bifida

## Abstract

Background: In the last twenty-five years, Onabotulinum Toxin A (BTX-A) has gained increasing popularity for neurogenic lower urinary tract dysfunction (NLUTD) treatment. To maintain its efficacy, repeated BTX-A intradetrusor injections are required over time, with unknown effects on the bladder wall in children. The aim of this paper is to report long-term effects on the bladder wall in children treated with BTX-A. Methods: Children with NLUTD not responsive to anticholinergics were treated with BTX-A, according to our protocol, with bladder wall control using endoscopic cold-cup biopsy. Specimens were evaluated considering edema, chronic inflammation, and fibrosis. Results: Of the 230 patients treated from 1997 to 2022, we considered only specimens obtained in patients who had received ≥5 treatments (36 children), considered as the threshold to evaluate clinical effectiveness on long-term treatment with BTX-A. Most of them had congenital NLUTD (25 patients) and detrusor overactivity (27 patients). In all, increased edema and chronic inflammation with reduced fibrosis over time was reported; these data were not statistically significant. No difference was observed between patients with congenital and acquired diseases. Conclusions: Repeated intradetrusor BTX-A injections are not related to significant histological alterations in children, similarly with adults, and repeated injections could be considered safe.

## 1. Introduction

The intradetrusor injection of Onabotulinum Toxin A (Botox^®^, Allergn Inc., Irvine, CA, USA), here defined as BTX-A, is currently one of the most popular therapeutic options for neurogenic lower urinary tract dysfunction (NLUTD) in children [1]. BTX-A is generally used in the case of resistance of poor tolerance to anticholinergic drugs. Clean intermittent catheterization (CIC) with anticholinergic represents the first-line treatment of NLUTD [1]. An early management with CIC and antimuscarinic medication of NLUTD led urologists to a consensus that these patients could not develop upper tract deterioration. In the past, there was concern regarding the failure of this treatment. For many years, the next therapeutic step was a reconstructive surgical procedure. Therefore, many children were subjected to bladder augmentation, while others, where surgical treatment was not performed or refused by the family, bladder fibrosis and upper urinary tract deterioration was often observed. For this reason, in the last twenty-five years, BTX-A has gained increasing popularity, even for the pediatric population, representing a minimally invasive treatment able to offer an alternative where medical treatment failed.

Several studies have evaluated the efficacy and safety of this therapy in children [1,2,3,4]. Intradetrusor injection of BTX-A leads to a significant improvement of continence, reducing maximum detrusor pressure, increasing maximum cystometric capacity, and ameliorating bladder compliance. This treatment seems to be more effective in bladders with detrusor overactivity, while non-compliant bladders without evident detrusor contractions are unlikely to respond to this treatment. In most published studies, the dose of BTX-A was 10 U/kg, up to a maximal dose of 300 U, involving 30 trigone-sparing injections of 10 U/kg/mL in the detrusor. More recently, a multicentric international phase III study recommended a dosage of 200 U, not exceeding 6 U/kg [5]. BTX-A seems to reach efficacy levels at 2 weeks and maximum effects within a month. The effect of BTX-A ranges from 3 to 8 months, and for this reason, repeated treatments are required over time. Repeated bladder injections of BTX-A have been demonstrated to be effective. Regarding the safety of this treatment, one of the major concerns is still the evolution of bladder fibrosis in children over time. Surprisingly, we found only one report evaluating the possible bladder histological alteration due to the repeated intradetrusor injections in the pediatric population [6]. The aim of our study is to present the long-term results of our prospective study, evaluating histological bladder wall modification over time in patients treated with BTX-A from infancy.

## 2. Materials and Methods

The study was approved by the Ethical Committee of our hospital (200602R001820).

Patients were enrolled after we obtained patients’ or parents/caregivers’ informed consent.

**Figure 1:**
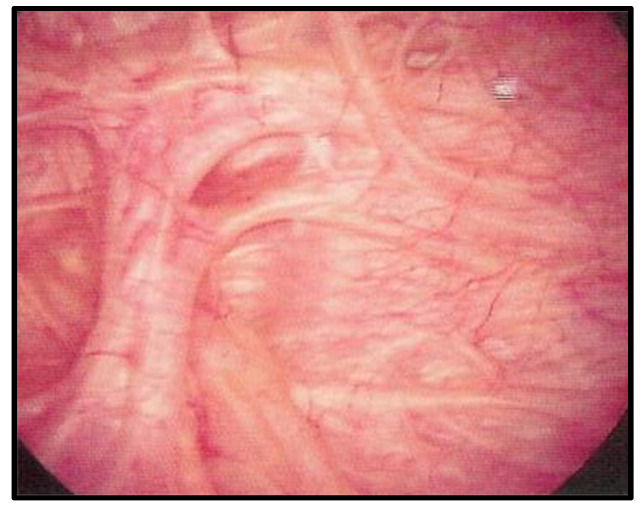
Neurogenic lower urinary tract dysfunction with trabeculae of the mucosa, in cystoscopic view.

Patients with neurogenic lower urinary tract dysfunction not responding to conventional first-line treatment were subjected to Onabotulinum Toxin A injections (Figure 1).

**Figure 2:**
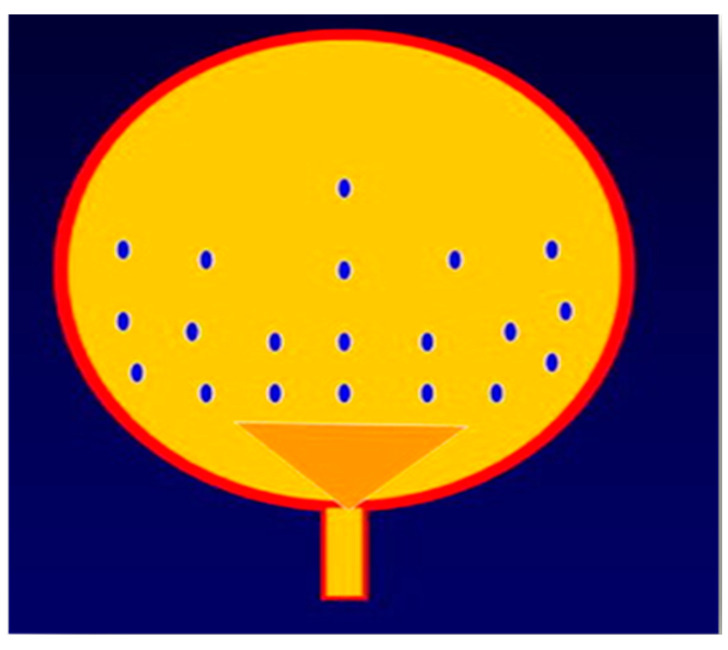
Bladder injection scheme.

As of 2003, we standardized our injection technique: 8 U/kg of BTX-A, using a rigid cystoscope and a flexible needle. Injection is always performed in the operating room and under general anesthesia. Multiple injections were administered following a virtual vertical line (as shown in Figure 2), starting 0.5–1 cm lateral and above the urethral orifice.

**Figure 3:**
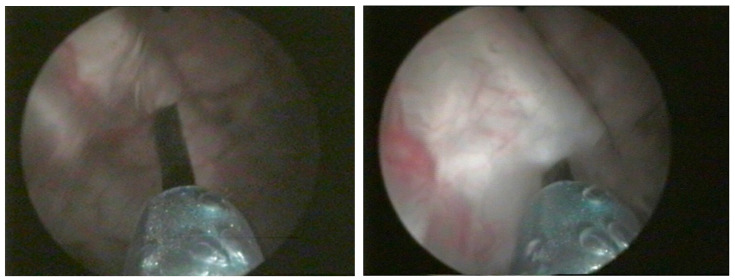
BTX-A injection with flexible collagen needle, into a trabecula.

Each injection was 1 mL (10 U/1 mL of saline solution), preferably into the trabeculae, with a maximum depth of 5 mm (Figure 3). Particular attention was paid to exclude trigone and blood vessels from the injection [7].

**Figure 4:**
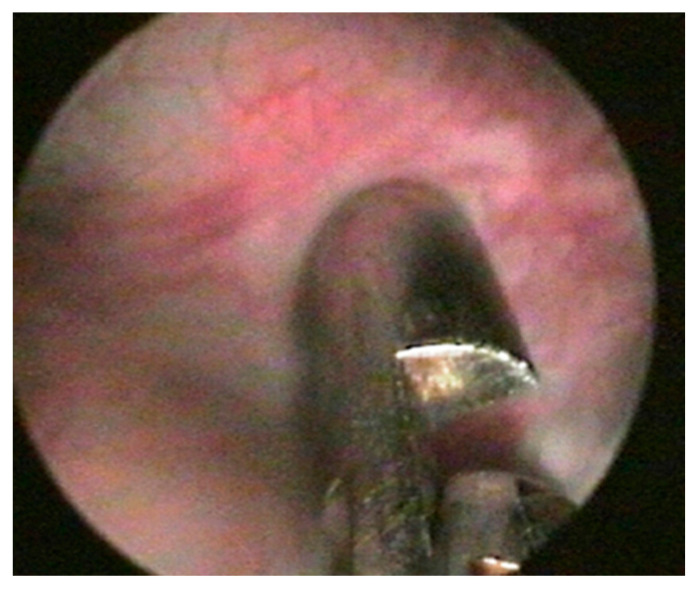
Bladder cold-cup biopsy during cystoscopy.

All patients underwent an endoscopic cold-cup biopsy (Figure 4) immediately before BTX-A injection, from the posterolateral bladder wall 1.5 to 2 cm above the ureteral orifice.

Specimens were fixed in 10% formalin and paraffine-embedded. Tissue sections were stained with Hematoxylin and Eosin (H&E) and Masson’s trichrome to assess fibrosis.

Edema, inflammation, and fibrosis were classified using a qualitative grading scale (0: none, 1: mild, 2: moderate, and 3: severe). Edema and inflammation were classified as mild (+), moderate (++), or severe (+++). Fibrosis was classified as mild (<20% of muscle layer or lamina propria), moderate (20–30%), or severe (>30%).

At the end of the procedure, a Foley catheter was placed and removed the following day, with the beginning of intermittent catheterization and subsequent discharge, with 5-days antibiotic therapy [7].

For statistical purposes, we considered biopsies collected at T1 (baseline, immediately before the first BTX-A injection), and during the 5th (T5), 6th (T6), and 7th (T7) BTX-A injections.

Fisher’s exact test was used to perform statistical analysis between qualitative variables. The Wilcoxon matched-pairs test and the nonparametric Mann–Whitney U test were used, respectively, for intragroup and intergroup comparisons. A *p*-value less than 0.05 was considered statistically significant.

Exclusion criteria: patients with less than 5 BTX-A treatments, those with an inadequate specimen for histological analysis, patients treated with BTX-A injection exclusively into the external urinary sphincter (due to detrusor external sphincter dyssynergia), and patients with incomplete clinical data.

All patients with symptomatic UTIs in the previous 3 months were also excluded from our analysis.

## 3. Results

A total of 230 children were treated with BTX-A in our hospital from 1997 to 2022.

We considered the repetition of at least 5 BTX-A treatments as clinical effectiveness and long-term therapy. We found 70/230 patients with a range of 5-22 BTX-A injections (treatment time-lapse: average 7.2 years, range 2.2–14.7 years). We included in the study only 36/70 children, considering only those with all specimens eligible for histological examination and all clinical data available.

The average age at first treatment with BTX-A was 5.6 years (range: 9 months–17.5 years).

Most of these children (25 patients) suffered from neurogenic lower urinary tract dysfunction due to a congenital disorder, and the other 11 were affected by acquired NLUTD (Table 1).

On urodynamic examination, performed and evaluated according to ICCS criteria [8], neurogenic detrusor overactivity (NDO) was observed in 27 patients, whereas the other 9 children had a high-pressure low-compliant bladder (LCB). All patients were on clean intermittent catheterization (CIC) regimen, and 15 of them had recurrent urinary tract infections before the first BTX-A injection. The mean age at first treatment was 5.6 years (range: 1.8–18.5 years), and the mean number of injections was 7.4 (range: 5–16 injections). BTX-A injections were repeated approximately every 12.3 months (range: 3.6–33.4 months).

Histological findings at baseline (immediately before the first BTX-A injection, T1) and during the 5th (T5), 6th (T6), and 7th (T7) treatments are reported in Table 2.

**Figure 5:**
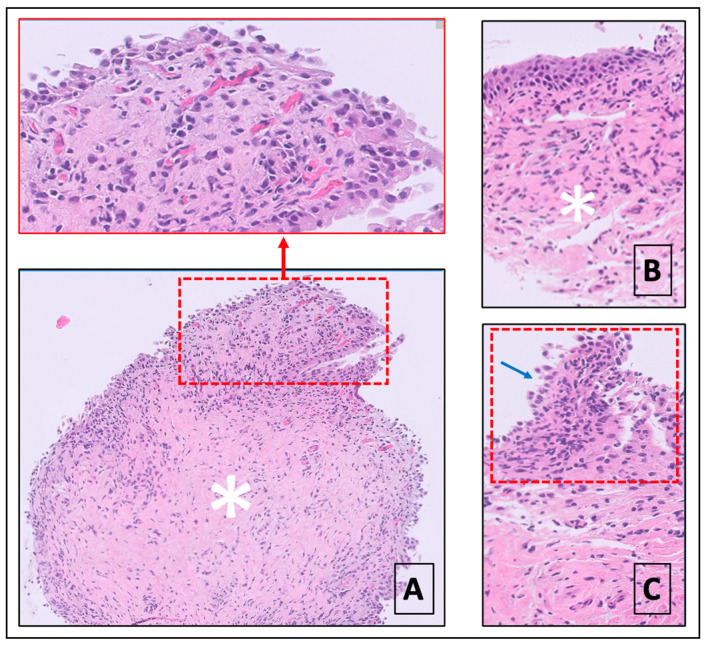
(**A**) Mild chronic inflammatory infiltrate and moderate fibrosis of bladder mucosa: inflammation shows a prevalent sub-epithelial distribution (dashed red line and red insert), whereas fibrosis extends deeper in the lamina propria (white asterisk). (**B**) Moderate interstitial fibrosis (white asterisk), without significant inflammation of the bladder mucosa. (**C**) Moderate inflammation (dashed red line) and transitional epithelium reactive hyperplasia (blue arrow). No interstitial fibrosis is observed.

All samples included urothelium and lamina propria. Inflammatory infiltration observed was typical of chronic inflammation (lymphocytic–plasma cell infiltrates), associated with granulocytes in three biopsies. Three biopsies showed follicular cystitis. Fibrosis, when detected, was localized in the lamina propria (Figure 5). Due to the nature of the biopsy (partial thickness), it was not possible to evaluate any smooth muscle layer alterations.

Edema, chronic inflammation, and fibrosis were identified at baseline in 20 (56%), 27 (75%) and 12 (33%) out of 36 patients, respectively. We found a higher percentage of biopsies positive for edema and chronic inflammation at T5 (biopsy performed immediately before the 5th injection). On the contrary, fibrosis seemed to be less frequent over time. However, these variations did not follow a linear trend and were not statistically significant (Table 2).

Histological findings were compared statistically between patients affected by congenital and acquired neurogenic lower urinary tract dysfunction (Table 3). Intragroup comparison showed no significant difference in terms of edema, inflammation, or fibrosis at T1 or after repeated treatment in the two groups. Sample size was too small to allow a sound statistical evaluation of intragroup edema, inflammation, or fibrosis score variation after repeated treatments (Wilcoxon matched-pairs test).

To investigate whether a specific urodynamic pattern could have a histological counterpart, patients were divided into two different groups on the basis of urodynamic parameters: NDO vs LCB. Data are reported in Table 4. Fibrosis was much more frequent both at baseline and after repeated treatment in patients suffering from low-compliant bladder compared with those affected by NDO. However, these results were not statistically significant.

## 4. Discussion

At present, the primary aim of NLUTD management is to provide the patient with the best long-term quality of life with preservation of normal renal function. Over time, knowledge on pathophysiology, pathogenesis, and treatment of different causes and patterns of NLUTD increased, even considering evidenced-based ways to manage these patients. Moreover, with the advent of CIC and anticholinergics, the renal function has been preserved without surgery, in many cases. Another important step in NLUTD management has been the urological use of BTX-A.

BTX-A is the most commonly used commercial form of neurotoxin produced by the bacterium Clostridium Botulinum, able to penetrate the membranes of neuronal cells. This toxin was originally thought to act solely by inhibiting the presynaptic release of acetylcholine, thus leading to relaxation of smooth and striated muscles. To date, it is known that the mechanism of action of this neurotoxin is much more complex. BTX-A can act on sensory and motor nerves, involving different neurotransmitters and neuropeptides; it can also inhibit the release of calcitonin gene-related peptide, decreasing suburothelial sensory receptors TRPV1 and P2X, controlling local inflammation, and reducing bladder sensation and pain, with good results in pain syndrome and interstitial cystitis in adult patients [2,9,10,11]. These actions of botulinum toxin, therefore, allow improvement of bladder function, evident by urodynamic examination with improvement of bladder capacity, compliance, detrusor overactivity, and bladder-filling pressure. Response to this therapy can be very heterogeneous, with an increase of maximum cystometric capacity from 27% to 162% of patients, an improvement in bladder compliance of 28–176%, and a decrease in maximum detrusor pressure of 32% to 54% [1].

The earliest data on the efficacy of BTX-A in the treatment of NLUTD date back to 2000, while the first report on the pediatric population was published by Schulte-Baukloh in 2002 [3].

BTX-A changed the management of NLUTD in children, allowing them to avoid or postpone reconstructive and invasive surgery to adolescence or adulthood.

NLUTD in children has been traditionally confined to spina bifida (SB) and other congenital spinal dysraphysm. Due to increased knowledge of neuro-urology, genetics, and metabolic disease and the improvement of imaging, many diseases potentially involving the function of lower urinary tract have been discovered. These led to a deeper comprehension of NLUTD pathophysiology. The knowledge of NLUTD in children is related mainly to spina bifida (open and closed), because other neurological diseases, such as spinal cord injury, iatrogenic injuries, tumor, or systemic disease, are considered to be rare diseases.

Children with SB and other spinal dysraphism (SBoD) often develop an NLUTD. SB and SBoD include different forms of neural tube defects, presenting in a large spectrum of clinical situations, including different levels of NLUTD. From a urological point of view, the most important distinction is between open and occult spina bifida. Open SB refers almost exclusively to myelomeningocele, where NLUTD are commonly observed from the birth and management is required for life-long. In the closed spina bifida, or occult spinal dysraphism (OSD), the NLUTD can appear with a different clinical spectrum related to bony defect, nerve roots anomalies, or with compression or stretch of the nervous structure. Among these “OSD”, many abnormalities can remain hidden for years; others can lead to clinical pathologies, such as tethered cord syndrome (TCS), which includes a series of symptoms and signs due to dysfunction of motor and sensory neurons. In TCS, the clinical pathology is the result of an abnormal tension on the spinal cord, with a thickening of the filum terminale and a low attachment of the spinal cord. These anomalies lead to a stretching of the nervous structures, which can manifest themselves in lower limb symptoms, musculoskeletal anomalies, neurogenic lower urinary tract dysfunction, and bowel dysfunction [12,13,14,15,16,17,18,19].

NLUTD can appear at birth or later in life, with urinary tract infections, failure to achieve continence, progressive hydronephrosis, change in micturition frequency, and urinary retention. Sometimes NLUTDs are transient and related to growth, as in puberty. In all these situations, the opportunity to have a conservative management, such as BTX-A, has been very successful, permitting patients to avoid major surgery in many cases [1,2,3,4,15,16,17].

Among the other causes of NLUTD, in addition to spinal dysraphism, are sacral agenesis, spinal cord injuries due to traumatic or oncological causes, and pathologies of the central nervous system [12,13,14]. In our sample, 70% of patients had a congenital cause of neurogenic lower urinary tract dysfunction.

In all these patients, urodynamic evaluation can be very useful, addressing them to a specific treatment according to the presence of detrusor overactivity (the most frequent pattern described), reduced bladder compliance with elevated intravesical pressures, detrusor sphincter dyssynergia, decreased sensation, or hypocontractility of the detrusor. These alterations in bladder function can lead to progressive damage of the urinary tract, up to renal failure, as previously mentioned, requiring dialysis or renal transplantation; for this reason, treatment should be started immediately.

The first approach is represented CIC, starting at birth in the most severe forms of NLUTD. From a pharmacological point of view, antimuscarinic therapy is the first line of therapy. These drugs can reduce or prevent detrusor hyperactivity and lower bladder pressures, with a success rate of up to 93% for oxybutynin (the most used drug of this category). Unfortunately, the presence of adverse effects limits its use or the duration of its administration. In the past, the only alternative solution for these situations was represented by surgery, with bladder augmentation (with ileum or colonic segments) often associated with urinary tract diversion. All these surgical procedures are associated with a risk of complications in the short or long term. To date, injection of Botulinum toxin represents a minimally invasive option before surgery. Therapy with BTX-A manifests its greatest success in the forms of NB with detrusor overactivity [1,14,19,20]. Of our 36 patients with NLUTD refractory as the first-line therapy and, therefore, subjected to BTX-A injections, 75% had a pattern of detrusor overactivity, and the remaining 25% had a low-compliance bladder. In all, BTX-A allowed them to avoid or postpone major surgical procedures. These patients with low-compliant bladder presented a histological pattern of fibrosis different from patients with NDO. Fibrosis was more frequent in patients suffering from low-compliant bladder. However, these differences were not statistically significant. Effectiveness of BTX-A in these patients could be explained by this point, with further studies needed on urothelium function.

The therapeutic effect of BTX-A varies over time. It can be seen within 2 weeks from the injection, with its maximum effectiveness in 4–6 weeks, and it can be effective for 3–12 months. Therefore, repeated injections are necessary to maintain the functional effect on the bladder wall [1,4]. In our experience, children were subjected to BTX-A injection approximately every 12 months, with a range between 3.6 and 33.4 months, depending on the duration of the therapeutic effect and adherence to therapy of the individual patient.

While this treatment is mainly performed with a flexible cystoscope, under local anesthesia or light sedation in the adult population, it is mainly performed under general anesthesia or sedation in the pediatric population [1,7,15,21].

Due to the need to perform multiple intradetrusor injections, the possible histological alterations of the bladder wall have attracted increasing attention. The concern was related to the risk of increasing fibrosis of the bladder wall with the repeated injections of BTX-A.

Fibrosis is known as a frequent finding in NLUTD, usually associated with mononuclear lymphocyte inflammation and concomitant edema [20].

Comperat et al. analyzed full-thickness bladder specimens, comparing the presence of bladder fibrosis in patients with neurologic detrusor overactivity treated with or without BTX-A; they highlighted the presence of less fibrosis in patients previously injected with Botulinum toxin [20]. Apostoliditis et al. confirmed the absence of increased fibrosis, inflammation, and dysplasia in the analysis of bladder biopsies of patients treated with single or limited BTX-A injections [22].

Jia et al. found a reduction in bladder fibrosis in rats with spinal cord injury (SCI) subjected to intradetrusor injection of BTX-A, likely due to a suppression of the transforming growth factor beta 1 (TGF-beta1), a pro-fibrosis cytokine [23]. Temeltas et al. reported the same results; BTX-A injections seemed to reduce fibrosis in rat models, regardless of the time interval between SCI and Botulinum therapy [24]. On the other hand, Bushnell et al. reported a better preservation of the bladder health in rats treated with BTX-A immediately after SCI, with a reduced development of fibrosis and hypertrophy, normally associated with SCI [25]. In our study, we compared the histological changes in patients with congenital or acquired neurogenic lower urinary tract dysfunction, without finding statistically significant differences; the same data emerged in the comparison between overactive and low-compliant bladders.

As we found in our previous study [6], repeated BTX-A injections do not lead to increased bladder fibrosis; moreover, comparing patients who had not received this therapy with those subjected to multiple BTX-A injections, fibrosis was less represented. In the present study, we confirmed these data. We evaluated the evolution of the histological changes in the same patient before starting therapy with BTX-A (T1) and after multiple injections, up to 6 treatments (T7, biopsy performed immediately before the 7th injection). Similarly, we found a progressive reduction of fibrosis with an increased number of treatments, although it was not statistically significant.

These positive data may be explained with better management of the NLUTD. On the contrary, however, a slight increase in edema and chronic inflammation emerged over time, which was also not statistically significant.

Despite its known clinical efficacy, a high discontinuation rate of BTX-A therapy is reported in the literature [26]. Baron et al. reported that over a mean of 100.9 months of follow-up, 64/140 adult patients abandoned this therapy after the 4^th^ treatment, while 51 patients were lost to follow-up. They found treatment failure to be the first cause of discontinuation (43.7% of cases); other causes were patient decision, increased urinary incontinence not related to drug injection, progression of neurological disease, and adverse events [27].

These data match with our experience, with only 70/230 patients undergoing at least 5 treatments over time. Several explanations were suggested for this phenomenon: preference for other forms of treatment and a progressive loss of clinical or urodynamic efficacy. Among the causes of the reduction of clinical response over time, the formation of neutralizing antibodies has been proposed; the presence of these antibodies is well known in patients treated for cervical dystonia or spasticity but not identified so clearly after intradetrusor treatment. Another theory was the progressive increase in bladder fibrosis due to the repeated injections, which could reduce the spread of the toxin in the bladder wall, reducing its effectiveness [24].

Following this second theory, we wondered whether any histological modifications could lead to a reduction in the effectiveness of BTX-A and, therefore, to the abandonment of the therapy. However, the presence of important histological alterations that could confirm our theory does not emerge from our data, and in our opinion, our histological evaluation could be useful to define the long-term effectiveness [25,28] and adherence to BTX-A treatment. This study is the first long-term evaluation of BTX-A effects on the bladder wall in children.

Some limitations are present in our study: first, this is a single-center experience. Second, the study was based on a protocol that, when established and approved, included only histological evaluation, so immunohistochemistry was not performed. Further studies on urothelium function in children after BTX-A repeated injections could be useful to elucidate some questions that are still open on the effectiveness of BTX-A, not well-related to the grade of fibrosis.

NLUTD significantly impacts quality of life for many children born with congenital diseases, such as spina bifida, or acquired bladder dysfunctions related to spinal cord injury due to road or sport trauma, as well as others related to iatrogenic injury by oncological, medical, or surgical treatment. For all these children, who currently are adults with a long life expectancy, minimally invasive treatments, such as BTX-A, are precious, permitting the treatment of low urinary tract dysfunction and symptoms, preserving the upper urinary tract, renal function, and avoiding surgical procedures. Although much has been learned about bladder function over the past decades, some unknowns remain to be clarified.

One of the major fields to investigate is urothelium function in NLUTD from childhood to adulthood: urothelium integration with neuronal signaling, immune interactions in urinary tract infections, disease outcome, and response to local treatment, such as BTX-A. Several studies have been conducted in adult populations to evaluate urothelium function in NLUTD [29,30] as well as on BTX-A effects on the bladder wall in adults [31], finding that BTX-A acts on the modulation of Adenosine triphosphate (ATP) and nitric oxide (NO) release from the urothelium and transient receptor potential vanilloid subfamily-1 (TRPV1), decreases P2X purinoceptor (P2X3), and reduces nerve growth factor content in bladder tissue.

Nonetheless, until now, these investigations are lacking in pediatric populations; thus, it would be useful to understand whether an early treatment with BTX-A could be able to avoid any grade of fibrosis that is commonly observed in all patients, even those treated from the birth with anticholinergic and CIC.

## 5. Conclusions

Despite the small sample examined and with the limitation of a single-center experience, we can conclude that in the pediatric population, repeated intradetrusor BTX-A injections do not to lead to histological alterations, similar to what has been observed in the adult population. According to these results, the safety of BTX-A is confirmed, and reduced effectiveness is not related to fibrosis. Further studies on urothelium function in pediatric populations could be useful to clarify BTX-A effectiveness in the long term.

This work is generated within the European Reference Network for Rare Urogenital Diseases and Complex Conditions (ERN eUROGEN).

## Figures and Tables

**Table 1:** Congenital and acquired causes of neurogenic lower urinary tract dysfunction.

Congenital Condition: 25 Patients		Number of Patients
	Myelomeningocele	6
	Lipomyelocele	3
	Caudal regression syndrome	2
	Occult spinal dysraphism	13
	Congenital disorders of glycosylation (CDG)	1
Acquired condition: 11 patients		
	Spinal cord injury	3
	Peripheral nerve lesions	1
	Central nervous system tumors	2
	Sacrococcygeal teratoma	3
	Neuroblastoma	2

**Table 2:** Edema, inflammation, and fibrosis score over time during BTX-A treatment (T1, T5, T6, and T7): **0**, none; **1**, mild; **2**, moderate; **3**, severe. No. of pts: number of patients; * Fisher’s exact test.

	T1	T5	T6	T7
Patients (number)	36	36	25	17
EDEMA score: No. of pts (%)	20 (56%)	24 (67%)	19 (76%)	13 (76%)
**0**	0	0	0	
**1**	15 (75%)	19 (79%)	19 (100%)	12 (92%)
**2**	3 (15%)	4 (17%)	0	1 (8%)
**3**	2 (10%)	1 (4%)	0	0
*p*-value (vs. T1) *		0.47	0.12	0.23
INFLAMMATION score: No. of pts (%)	27 (75%)	32 (89%)	19 (76%)	15 (88%)
**0**	0	0	0	
**1**	20 (74%)	22 (69%)	17 (89%)	13 (87%)
**2**	5 (19%)	9 (28%)	2 (11%)	2 (13%)
**3**	2 (7%)	1 (3%)	0	0
*p*-value (vs. T1) *		0.22	1	0.47
FIBROSIS score: No. of pts (%)	12 (33%)	8 (22%)	8 (32%)	5 (29%)
**0**	0	0	0	
**1**	10 (83%)	8 (100%)	8 (100%)	5 (100%)
**2**	2 (17%)	0	0	0
**3**	0	0	0	0
*p*-value (vs. T1) *		0.43	1	1

**Table 3:** Edema, inflammation, and fibrosis over time during BTX-A treatment (T1, T5, T6, and T7) in patients with congenital and acquired neurogenic lower urinary tract dysfunction. No. of pts: number of patients; * Fisher’s exact test.

	Congenital				Acquired			
	T1	T5	T6	T7	T1	T5	T6	T7
Patients (number)	25	25	17	12	11	11	8	5
EDEMA: No. of pts (%)	12 (48%)	19 (76%)	14 (82%)	10 (83%)	8 (73%)	5 (45%)	5 (63%)	3 (60%)
*p*-value (vs. T1) *		0.08	0.05	0.07		0.39	1	1
*p*-value (vs. CONGENITAL) *					0.28	0.12	0.34	0.54
INFLAMMATION: No. of pts (%)	25 (70%)	23 (90%)	13 (76%)	11 (92%)	10 (91%)	9 (82%)	6 (75%)	4 (80%)
*p*-value (vs. T1) *		0.07	0.73	0.22		1	0.55	0.54
*p*-value (vs. CONGENITAL) *					0.22	0.57	1	0.51
FIBROSIS: No. of pts (%)	9 (36%)	5 (20%)	5 (29%)	3 (25%)	3 (27%)	2 (18%)	3 (38%)	2 (40%)
*p*-value (vs. T1) *		0.35	0.75	0.71		1	1	1
*p*-value (vs. CONGENITAL) *					0.71	1	1	0.60

**Table 4:** Edema, inflammation, and fibrosis over time, during BTX-A treatment (T1, T5, T6, T7) in patients with neurogenic detrusor overactivity (NDO) vs. high-pressure low-compliant bladder (LCB) at urodynamic study. No of pts: number of patients; * Fisher’s exact test.

	NDO				LCB			
	T1	T5	T6	T7	T1	T5	T6	T7
Patients (n)	27	27	20	12	9	9	5	5
EDEMA: No. of pts (%)	15 (56%)	19 (70%)	15 (75%)	11 (92%)	5 (56%)	5 (56%)	4 (80%)	2 (40%)
*p*-value (vs. T1) *		0.4	0.23	0.03		1	0.58	1
*p*-value (vs. NDO) *					1	0.44	1	0.05
INFLAMMATION: No. of pts (%)	21 (78%)	25 (93%)	15 (75%)	12 (100%)	6 (67%)	7 (78%)	4 (80%)	3 (60%)
*p*-value (vs. T1) *		0.75	1	0.15		1	1	1
*p*-value (vs. NDO) *					0.66	1	1	0.07
FIBROSIS: No. of pts (%)	8 (30%)	5 (19%)	5 (25%)	4 (33%)	4 (44%)	3 (33%)	3 (60%)	1 (20%)
*p*-value (vs. T1) *		0.53	0.76	1		1	1	1
*p*-value (vs. NDO) *					0.44	0.38	0.28	0.59

## Data Availability

Not applicable.
